# Quality of Life After Modified Radical Mastectomy (MRM): A Prospective Study Using the European Organisation for Research and Treatment of Cancer (EORTC) QLQ-C30 and QLQ-BR45

**DOI:** 10.7759/cureus.117585

**Published:** 2026-10-07

**Authors:** Gaurav Gupta, Tanushree Trivedi, Sangeeta Gupta, Dharmendra Kumar Pipal, Ravi Gupta, Mukul Singh, Rama S Rath, Manoj Prithviraj, Hari Kesh Yadav, Rajnish Kumar, Shahnawaz Ahmed Chowdhary, Manish Kumar, Jagani Harsha Sai, Raja Murtaza, Darshan Jalwal, Rajesh Kannan

**Affiliations:** 1 General Surgery, All India Institute of Medical Sciences, Gorakhpur, Gorakhpur, IND; 2 Physiology, All India Institute of Medical Sciences, Gorakhpur, Gorakhpur, IND; 3 Community Medicine, All India Institute of Medical Sciences, Gorakhpur, Gorakhpur, IND; 4 Psychiatry, All India Institute of Medical Sciences, Gorakhpur, Gorakhpur, IND

**Keywords:** eortc qlq-br45, eortc qlq-c30, modified radical mastectomy (mrm), observational study, quality-of-life

## Abstract

Background: Modified radical mastectomy (MRM) remains a frequently performed operation for breast cancer, particularly where breast conservation is not feasible. Although it achieves reliable locoregional control, MRM is associated with lasting effects on physical function, body image, and psychosocial well-being. As survival improves, patient-reported quality of life (QoL) has become an essential survivorship outcome. This study assessed QoL after MRM using the European Organisation for Research and Treatment of Cancer Quality of Life Questionnaire-Core 30 (EORTC QLQ-C30) and the breast-specific QLQ-BR45, complemented by the Unconditional Self-Acceptance Questionnaire (USAQ), and examined how QoL domains are associated with patient age.

Methods: In this prospective observational study, 70 women who underwent MRM for invasive breast carcinoma at a tertiary-care institute were followed serially (diagnosis, postoperative Day 1, Day 7, one month, and six months); QoL questionnaires were completed at the six-month visit and scored according to the EORTC scoring manuals. Scores were compared across five age strata (≤39, 40-49, 50-59, 60-69, and ≥70 years) using one-way analysis of variance (ANOVA) with eta-squared (η^2^), confirmed with the Kruskal-Wallis test, and adjusted for multiple testing (Benjamini-Hochberg false discovery rate, or FDR). Domains that differed by age were examined with multivariable regression adjusting for stage, neoadjuvant chemotherapy, and radiotherapy.

Results: The mean age was 51.59±11.71 years; most women had T2 (n=48, 68.6%), node-negative (n=39, 55.7%), non-metastatic (n=67, 95.7%) disease. Severe postoperative pain occurred in 98.6% (n=69), and the mean hospital stay was 13.66±2.26 days. The QLQ-C30 summary score was 74.34±11.84 and the USAQ score 76.79±4.05. After FDR correction on both ANOVA and Kruskal-Wallis testing, fatigue (η^2^=0.21), appetite loss (η^2^=0.18), body image (η^2^=0.49), future perspective (η^2^=0.22), arm (η^2^=0.19), breast (η^2^=0.18), endocrine therapy (η^2^=0.23), and endocrine sexual (η^2^=0.52) symptoms differed by age. Women aged 49 years or younger had poorer body image, whereas symptom scores were higher from age 50. All 11 women aged ≤39 years and 10 of 19 aged 40-49 years were sexually active, versus none of the 40 women aged 50 years or above. Age remained associated with all eight scales after adjustment, and stage III-IV disease was independently associated with poorer global health status (β=-8.29, p=0.004). USAQ scores did not vary with age (p=0.106).

Conclusions: In this single-centre cohort, QoL six months after MRM was multidimensional and associated with age and disease stage. Younger women reported poorer body image and future perspective, while older women and those with advanced disease reported a heavier symptom burden. These exploratory findings suggest that body image, sexual health, arm morbidity, and symptom control may warrant attention in post-mastectomy care, and they need confirmation in larger studies.

## Introduction

Breast cancer is the most commonly diagnosed malignancy among women worldwide, with an estimated 2.26 million new cases annually according to the Global Cancer Observatory (GLOBOCAN) 2020 [1]. In India, it has overtaken cervical cancer to become the leading cancer among women; the National Cancer Registry Programme estimated 216,108 new cases in 2022, a crude incidence rate of 30.4 per 100,000, and a cumulative lifetime risk of approximately 1 in 29 women [2]. Because earlier detection and multimodal treatment have improved survival, the population of long-term survivors continues to grow, and the enduring physical and psychosocial consequences of treatment have become central to survivorship care [3].

Surgery remains the cornerstone of curative management, and modified radical mastectomy (MRM) continues to be widely performed, particularly in low- and middle-income settings and for patients with locally advanced, multifocal, or centrally located disease, or when breast conservation and reconstruction are not feasible. While MRM offers reliable oncological clearance, it entails removal of the entire breast together with axillary nodal clearance, producing substantial anatomical and functional change. Reported sequelae include persistent post-mastectomy pain, restricted shoulder mobility, sensory disturbance, and lymphedema, alongside altered body image, sexual dysfunction, and emotional distress [4,5]. These effects extend the impact of treatment well beyond survival and can be especially pronounced in younger women and those in active social or occupational roles.

Quality of life (QoL) is a multidimensional construct encompassing physical, functional, emotional, and social well-being, and is now recognised as a key patient-reported outcome after cancer treatment. The European Organisation for Research and Treatment of Cancer (EORTC) Quality of Life Questionnaire-Core 30 (QLQ-C30) is a validated, generic instrument covering global health status, five functional scales, and several symptom scales [6]. Its breast-specific companion, the QLQ-BR45, is an updated and expanded module that succeeds the earlier BR23 and adds domains particularly relevant to contemporary treatment, including endocrine therapy, skin mucositis, and endocrine sexual symptoms [7]. Together, these instruments allow a comprehensive appraisal of survivorship experience, which can be further complemented by measures of psychological adjustment such as the Unconditional Self-Acceptance Questionnaire (USAQ) [8].

Despite growing attention to survivorship, comprehensive QoL data after MRM using the newer QLQ-BR45 remain limited, especially from Indian tertiary care settings, and most available studies capture QoL during or shortly after treatment without structured, age-stratified evaluation [9,10]. The primary objective of this study was to assess QoL six months after MRM using the EORTC QLQ-C30 and QLQ-BR45. The secondary objectives were to compare QoL domains across age groups in order to identify domains that may need targeted supportive care, and to assess unconditional self-acceptance using the USAQ. A further secondary objective was to examine associations between clinical variables and QoL. The primary analysis is a cross-sectional assessment at the six-month visit, not a longitudinal analysis of change over time; the earlier serial visits recorded clinical and postoperative recovery data, which are reported descriptively. The USAQ was included because the EORTC instruments measure symptoms and functioning but not psychological adjustment, and unconditional self-acceptance may shape how women cope with the loss of a breast regardless of symptom burden.

## Materials and methods

Study design and setting

This prospective observational study was conducted in the Department of General Surgery of a tertiary care teaching institute over a 12-month recruitment period. Consecutive eligible women undergoing MRM for breast cancer were enrolled and followed serially. The study was approved by the Institutional Human Ethics Committee (IHEC/AIIMS-GKP/BMR/305/2024) and was performed in accordance with the principles of the Declaration of Helsinki. Written informed consent was obtained from every participant before enrolment.

Female patients with histopathologically confirmed invasive carcinoma of the breast of any clinical stage who were scheduled to undergo MRM were eligible and were enrolled prospectively at diagnosis, before surgery. Patients were excluded if they were pregnant, declined consent, or had severe psychiatric illness, cognitive impairment, or any condition that precluded reliable completion of the questionnaires. A total of 70 women fulfilled the eligibility criteria and were included. No formal a priori sample-size calculation was performed; the sample comprised all eligible women presenting during the recruitment period. A post hoc sensitivity analysis showed that, with 70 participants across five groups, α=0.05 and 80% power, the study could detect an effect of Cohen's f≈0.43 (η^2^≈0.15) or larger; smaller age effects may therefore have been missed.

Assessments and instruments

Tumour stage was recorded per the American Joint Committee on Cancer (AJCC) eighth edition [11], radiological findings per the Breast Imaging Reporting and Data System (BI-RADS) lexicon [12], molecular subtype per the 2011 St. Gallen consensus criteria [13], and preoperative anaesthetic risk per the American Society of Anesthesiologists (ASA) physical status classification. Oestrogen receptor and progesterone receptor status were determined by immunohistochemistry per the American Society of Clinical Oncology/College of American Pathologists (ASCO/CAP) guideline [14], and human epidermal growth factor receptor 2 (HER2) status per the ASCO/CAP HER2 testing guideline [15]. Each participant was assessed at fixed visits: diagnosis (baseline), postoperative Day 1, Day 7, one month, and six months. The QoL questionnaires analysed in this report were completed once per patient at the six-month postoperative follow-up visit (approximately six months after surgery). The earlier visits recorded clinical and postoperative recovery data. The six-month visit was chosen for the QoL analysis because it is a defined point after the acute postoperative period at which all age groups were at the same stage of follow-up. All 70 enrolled women completed the six-month assessment, so there was no loss to follow-up. Structured working proformas captured demographic, clinicopathological, and treatment variables, together with postoperative recovery parameters (Visual Analogue Scale (VAS) pain score [16], Southampton wound-grade classification of surgical-site infection [17], and duration of hospital stay). Postoperative analgesia followed the standard institutional protocol, and the VAS score was recorded on the day of surgery.

QoL was evaluated using three validated instruments. The EORTC QLQ-C30 (version 3.0) comprises 30 items assessing global health status, five functional scales (physical, role, emotional, cognitive, and social), and symptom scales; responses are transformed to a standardised 0-100 scale, with higher functional and global scores indicating better QoL and higher symptom scores indicating greater burden [6]. The EORTC QLQ-BR45 is a breast-cancer-specific module used alongside the QLQ-C30, scored on the same 0-100 metric across functional and symptom domains [7]. The USAQ, developed by Chamberlain and Haaga, comprises 20 items on a seven-point scale; the 11 negatively worded items were reverse-scored before summation, yielding a total score of 20-140, in which higher values denote greater unconditional self-acceptance [8]. All questionnaires were scored according to the respective EORTC scoring manuals and the USAQ scoring instructions [8,18]. All scales were calculated from the item-level responses according to the EORTC scoring manuals [18]. QLQ-BR45 scales followed the published item structure [7]: body image (items 39-42), future perspective (43), sexual functioning (44-45), sexual enjoyment (46), breast satisfaction (74-75), systemic therapy side effects (31-34 and 36-38), upset by hair loss (35), arm symptoms (47-49), breast symptoms (50-53), endocrine therapy symptoms (54-56 and 63-69), skin mucositis symptoms (57-62), and endocrine sexual symptoms (70-73). For sexual functioning, sexual enjoyment, and breast satisfaction, a higher raw response indicates better function, so these scales were transformed without reversal, as specified in the scoring manual. Sexual enjoyment was scored only for sexually active women and upset by hair loss only for women who had lost hair. A woman was classed as sexually active if she answered item 45 ("Have you been sexually active?") with any response other than "not at all". The QLQ-C30 summary score was calculated as the mean of 13 QLQ-C30 scales, excluding global health status and financial difficulties, with symptom scales reversed [19]. All questionnaires were fully completed, with no missing items. The English versions of the EORTC QLQ-C30 and QLQ-BR45 were used, with permission obtained from the EORTC Quality of Life Group, and the English version of the USAQ was used. For women who could not read English, the authors translated the items verbally into the patient's own language; these translations were not formally validated. Questionnaires were self-completed, with an investigator reading the items aloud where literacy was limited.

Statistical analysis

Data were entered in Microsoft Excel (Microsoft Corp., Redmond, WA, USA) and analysed with IBM SPSS Statistics for Windows, Version 29 (Released 2022; IBM Corp., Armonk, New York, United States); the analyses added during revision were performed on the same patient-level data in Python (SciPy; Python Software Foundation, Fredericksburg, VA, USA). Categorical variables were summarised as frequencies and percentages, and continuous variables as mean ± standard deviation (SD). Categorical associations were tested using the Pearson chi-square test, with Fisher's exact test (or its Fisher-Freeman-Halton extension) applied where expected cell counts were low; for sexual activity across age groups, an exact p-value was obtained by Monte Carlo simulation (20,000 permutations). QoL scores were compared across the five age strata (≤39, 40-49, 50-59, 60-69, and ≥70 years; conventional decade bands with open-ended groups at the extremes) using one-way analysis of variance (ANOVA), with eta-squared (η^2^) as the effect size. Because EORTC scores are ordinal and several were skewed, every comparison was repeated with the Kruskal-Wallis test, and Welch's ANOVA was used as a further check for unequal variances. P-values from the 25 domain-level comparisons were adjusted using the Benjamini-Hochberg false discovery rate (FDR) method, and a domain was regarded as differing by age only if the q-value was below 0.05 on both ANOVA and Kruskal-Wallis testing; the Holm-Bonferroni correction was applied as a more conservative check. Pairwise differences were examined with the Games-Howell test, which does not assume equal variances or group sizes. Sexual functioning, sexual enjoyment, and upset by hair loss were reported descriptively, because the first had zero variance in three strata and the other two applied only to subgroups. To account for confounding, domains that differed by age were analysed with multivariable linear regression including age as a continuous variable (per 10 years), stage (III-IV vs I-II), neoadjuvant chemotherapy, and radiotherapy, so that the adjusted estimates do not depend on the small oldest stratum; all patients received adjuvant chemotherapy, and menopausal status and comorbidity were not recorded separately. Associations between clinical variables (stage, neoadjuvant chemotherapy, radiotherapy, Southampton wound grade III-V vs I-II, length of hospital stay, and receptor group) and key QoL scales were examined with the Mann-Whitney U test, Spearman correlation, or Kruskal-Wallis test, with FDR adjustment across these 48 tests. A two-sided p-value of less than 0.05 was considered statistically significant.

Given the sample size, the small oldest stratum (n=4), and the number of comparisons, all age-related comparisons are regarded as exploratory, and the findings are described as age-associated rather than as effects of age.

## Results

Seventy women who underwent MRM for invasive breast carcinoma were studied. The mean age was 51.59±11.71 years (range, 30-73). The largest age stratum was 50-59 years (n=21, 30.0%), followed by 40-49 years (n=19, 27.1%) and 60-69 years (n=15, 21.4%); women aged ≤39 and ≥70 years comprised 15.7% (n=11) and 5.7% (n=4) of the cohort, respectively. Body mass index was broadly comparable across age groups (approximately 24-25 kg/m^2^), and most patients were classified as ASA physical status II, indicating mild systemic disease. The baseline demographic and tumour characteristics are summarised in Table 1.

**Table 1:** Baseline demographic, tumour, and treatment characteristics and early postoperative recovery (n=70) ER: estrogen receptor; PR: progesterone receptor; HER2: human epidermal growth factor receptor 2; BI-RADS: Breast Imaging Reporting and Data System; AJCC: American Joint Committee on Cancer; VAS: Visual Analogue Scale; MRM: modified radical mastectomy

Characteristic	n	%
Age group		
≤39 years	11	15.7
40-49 years	19	27.1
50-59 years	21	30.0
60-69 years	15	21.4
≥70 years	4	5.7
Stage group (AJCC eighth edition)		
Early/operable (I-II)	50	71.4
Locally advanced (III)	17	24.3
Metastatic (IV)	3	4.3
T category		
T1	2	2.9
T2	48	68.6
T3	11	15.7
T4	9	12.9
Nodal (N) status		
N0	39	55.7
N1	23	32.9
N2	8	11.4
Metastatic (M) status		
M0	67	95.7
M1	3	4.3
Receptor status		
ER positive	42	60.0
PR positive	37	52.9
HER2 positive	25	35.7
Molecular subtype (2011 St. Gallen criteria)		
Luminal A	21	30.0
Luminal B (HER2-)	11	15.7
Luminal B (HER2+)	10	14.3
HER2-enriched	15	21.4
Triple-negative	13	18.6
BI-RADS category		
BI-RADS 1-3	4	5.7
BI-RADS 4	27	38.6
BI-RADS 5	39	55.7
Histological type		
Infiltrating ductal carcinoma	68	97.1
Infiltrating lobular carcinoma	1	1.4
Infiltrating mucinous carcinoma	1	1.4
Treatment received		
Modified radical mastectomy	70	100.0
MRM laterality: right	42	60.0
MRM laterality: left	28	40.0
Adjuvant chemotherapy	70	100.0
Neoadjuvant chemotherapy	25	35.7
Radiotherapy	32	45.7
Postoperative pain severity (VAS, day of surgery)		
Severe (7-10)	69	98.6
Moderate (4-6)	1	1.4
Duration of hospital stay		
≤10 days	7	10.0
11-14 days	43	61.4
≥15 days	20	28.6
Wound status (Southampton grade)		
Minor changes (I-II)	52	74.3
Moderate infection (III)	5	7.1
Severe complications (IV-V)	13	18.6

All 70 women underwent MRM and received adjuvant chemotherapy; MRM was right-sided in 60.0% and left-sided in 40.0%. Neoadjuvant chemotherapy was administered to 35.7% and radiotherapy to 45.7%. Early postoperative morbidity was pronounced: 98.6% of patients reported severe pain (VAS 7-10) on the day of surgery (mean VAS 7.83 ± 0.54), and the mean hospital stay was 13.66±2.26 days (range, 10-22), with only 10.0% discharged within 10 days. Wound assessment by Southampton grade showed predominantly minor changes (grades I-II) in 74.3%, moderate infection (grade III) in 7.1%, and severe complications (grades IV-V) in 18.6%. Treatment exposure and postoperative recovery parameters are also shown in Table 1.

Overall and age-wise QoL scores

Across the whole cohort, the QLQ-C30 summary score was 74.34±11.84, global health status/QoL was 53.33±9.97, and the USAQ total score was 76.79±4.05 (range 20-140), reflecting moderate overall QoL and a mid-range level of self-acceptance despite the physical burden of surgery. The summary score was lower in women aged 50-59 and ≥70 years (69.39±13.96 and 64.01±11.48) than in those aged ≤39 years (80.59±9.68; ANOVA p=0.021, Kruskal-Wallis p=0.066; this difference was therefore not confirmed on non-parametric testing). Domain-level results are given in Tables 2-3.

**Table 2:** EORTC QLQ-C30 scale scores across age groups Values are means ± SD on a 0-100 scale, calculated from item-level data according to the EORTC scoring manual. Test: one-way ANOVA; F(4,65), p-value, eta-squared (η^2^), and q (Benjamini-Hochberg FDR-adjusted p-values across 25 domain-level comparisons for ANOVA and for the Kruskal-Wallis test, shown as ANOVA/KW). *Significant after FDR correction on both ANOVA and Kruskal-Wallis testing (q<0.05). Pairwise (Games-Howell), Welch's ANOVA, and Holm-adjusted results are described in the text. The summary score is the mean of 13 QLQ-C30 scales, excluding global health status and financial difficulties, with symptom scales reversed [19]; it was not included in the FDR set. Higher functional, global, and summary scores indicate better QoL; higher symptom scores indicate greater burden. KW: Kruskal-Wallis; EORTC: European Organisation for Research and Treatment of Cancer; QoL: quality of life; ANOVA: analysis of variance; FDR: false discovery rate

Domain	≤39	40-49	50-59	60-69	≥70	F(4,65)	p	η^2^	q (ANOVA/KW)
Functional scales									
Physical functioning	90.91±12.74	83.16±16.39	74.92±20.54	75.56±15.05	63.33±17.64	2.91	0.028	0.15	0.071/0.068
Role functioning	83.33±16.67	77.19±16.86	65.08±24.10	66.67±16.67	58.33±16.67	2.72	0.037	0.14	0.071/0.090
Emotional functioning	47.73±16.28	48.25±15.11	52.78±13.00	58.33±8.33	60.42±7.98	1.95	0.113	0.11	0.141/0.102
Cognitive functioning	71.21±18.40	74.56±15.08	65.08±18.93	71.11±11.73	54.17±8.33	1.85	0.131	0.10	0.156/0.133
Social functioning	42.42±20.23	43.86±17.75	52.38±16.06	60.00±12.28	54.17±15.96	2.77	0.034	0.15	0.071/0.037
Symptom scales									
Fatigue*	21.21±17.54	28.65±18.26	43.92±19.40	32.59±15.41	50.00±19.25	4.22	0.004	0.21	0.021/0.034
Nausea and vomiting	0.00±0.00	10.53±20.19	19.84±24.51	8.89±16.51	29.17±34.36	2.57	0.046	0.14	0.077/0.090
Pain	9.09±15.57	7.89±14.02	23.81±21.46	12.22±16.02	29.17±20.97	3.15	0.020	0.16	0.055/0.068
Dyspnoea	3.03±10.05	1.75±7.65	12.70±16.59	8.89±15.26	16.67±19.25	2.42	0.057	0.13	0.089/0.098
Insomnia	27.27±20.10	26.32±17.84	41.27±17.97	33.33±17.82	41.67±16.67	2.22	0.076	0.12	0.112/0.113
Appetite loss*	3.03±10.05	10.53±15.92	17.46±17.06	11.11±16.27	33.33±0.00	3.59	0.011	0.18	0.034/0.037
Constipation	21.21±16.82	33.33±11.11	36.51±17.97	31.11±8.61	41.67±16.67	2.57	0.046	0.14	0.077/0.090
Diarrhoea	3.03±10.05	8.77±15.08	12.70±16.59	2.22±8.61	16.67±19.25	1.97	0.109	0.11	0.141/0.142
Financial difficulties	63.64±10.05	61.40±12.49	58.73±14.55	55.56±16.27	58.33±16.67	0.66	0.625	0.04	0.625/0.613
Global health status/QoL	56.82±10.42	53.51±9.34	51.19±10.96	55.56±6.80	45.83±14.43	1.36	0.258	0.08	0.280/0.351
QLQ-C30 summary score	80.59±9.68	76.86±9.95	69.39±13.96	76.25±8.91	64.01±11.48	3.10	0.021	0.16	-

**Table 3:** EORTC QLQ-BR45 domain scores across age groups Values are means ± SD on a 0-100 scale, calculated from item-level data according to the EORTC scoring manual; QLQ-BR45 scales follow the published item structure [7]. Test: one-way ANOVA; F(4,65), p-value, eta-squared (η^2^), and q (Benjamini-Hochberg FDR-adjusted p-values across 25 domain-level comparisons for ANOVA and for the Kruskal-Wallis test, shown as ANOVA/KW). *Significant after FDR correction on both ANOVA and Kruskal-Wallis testing (q<0.05). ^#^Reported descriptively only: sexual functioning had zero variance in three strata; sexual enjoyment was scored only for sexually active women (n=21) and upset by hair loss only for women who had lost hair (n=24). Higher functional scores (including sexual functioning, sexual enjoyment, and breast satisfaction) indicate better functioning; higher symptom scores indicate greater burden. ^Exact Monte Carlo chi-square test. KW: Kruskal-Wallis; EORTC: European Organisation for Research and Treatment of Cancer; ANOVA: analysis of variance; FDR: false discovery rate

Domain	≤39	40-49	50-59	60-69	≥70	F(4,65)	p	η^2^	q (ANOVA/KW)
Functional scales									
Body image*	43.94±9.20	49.12±8.28	59.52±10.96	67.22±5.86	64.58±7.98	15.58	<0.001	0.49	<0.001/<0.001
Sexual functioning^#^	43.94±15.41	19.30±17.80	0.00±0.00	0.00±0.00	0.00±0.00	-	-	-	-
Sexual enjoyment^#^	39.39±13.48	36.67±10.54	-	-	-	-	-	-	-
Sexually active, n (%)	11/11 (100.0)	10/19 (52.6)	0/21 (0.0)	0/15 (0.0)	0/4 (0.0)	-	<0.001^	-	-
Future perspective*	48.48±17.41	35.09±13.49	44.44±19.25	57.78±15.26	58.33±16.67	4.63	0.002	0.22	0.015/0.028
Breast satisfaction	36.36±10.05	34.21±8.74	36.51±6.71	40.00±12.28	41.67±16.67	0.99	0.420	0.06	0.438/0.357
Symptom scales									
Systemic-therapy side effects	12.55±17.60	21.55±20.35	34.69±23.09	21.27±17.16	35.71±22.17	2.77	0.034	0.15	0.071/0.034
Breast symptoms*	6.06±10.60	10.96±13.34	23.41±18.56	17.78±12.14	29.17±28.46	3.57	0.011	0.18	0.034/0.037
Arm symptoms*	8.08±14.13	14.04±13.78	26.98±18.11	18.52±11.63	33.33±30.09	3.86	0.007	0.19	0.029/0.037
Endocrine-therapy symptoms*	10.30±11.20	17.72±10.54	25.71±12.16	25.11±9.99	28.33±11.06	4.88	0.002	0.23	0.014/0.034
Endocrine sexual symptoms*	24.24±11.46	35.53±16.86	60.32±11.15	50.00±10.91	45.83±8.33	17.69	<0.001	0.52	<0.001/<0.001
Skin-mucositis symptoms	9.09±14.11	11.99±13.00	17.20±14.04	17.78±11.92	26.39±20.97	1.71	0.159	0.10	0.181/0.157
Upset by hair loss^#^	55.56±50.92	61.11±32.77	62.96±11.11	66.67±27.22	66.67±0.00	-	-	-	-

EORTC QLQ-C30 domain scores

On the QLQ-C30 (Table 2), physical, role, and social functioning differed by age on ANOVA (p=0.028, p=0.037, and p=0.034), with physical and role functioning declining and social functioning improving with age, but none of these remained significant after FDR correction. Among symptom scales, fatigue (p=0.004, q=0.021, η^2^=0.21) and appetite loss (p=0.011, q=0.034, η^2^=0.18) remained significant after correction and on Kruskal-Wallis testing (q=0.034 and q=0.037). On Games-Howell testing, fatigue was higher at 50-59 than at ≤39 years (mean difference 22.7 points, p=0.021), and appetite loss was higher at 50-59 than at ≤39 years (14.4 points, p=0.040); comparisons involving the ≥70-year group (n=4) were unstable because all four women in that group had the same appetite-loss score. Nausea and vomiting, pain, and constipation were significant before but not after correction. Global health status/QoL did not differ significantly by age (p=0.258), although mean scores ranged from 45.83 to 56.82 (η^2^=0.08); this spread of about 11 points is close to the threshold commonly regarded as clinically meaningful on the QLQ-C30 (about 10 points), and the study had only about 43% power to detect an effect of this size. Financial difficulties remained high across all strata (p=0.625).

EORTC QLQ-BR45 domain scores

On the QLQ-BR45 (Table 3), six scales differed by age after FDR correction on both ANOVA and Kruskal-Wallis testing: body image (η^2^=0.49), future perspective (η^2^=0.22), arm symptoms (η^2^=0.19), breast symptoms (η^2^=0.18), endocrine therapy symptoms (η^2^=0.23), and endocrine sexual symptoms (η^2^=0.52). Body image was poorest among women aged ≤39 years (43.94±9.20) and best at 60-69 years (67.22±5.86); on Games-Howell testing, it was poorer at ≤39 and 40-49 years than at 50-59 and 60-69 years (all p≤0.013) and poorer at ≤39 than at ≥70 years (p=0.028). Future perspective was lowest at 40-49 years and lower than at 60-69 years (p=0.001). Arm and breast symptoms were higher at 50-59 than at ≤39 years (p=0.025 and p=0.017), and endocrine therapy symptoms were higher at 50-59 and 60-69 than at ≤39 years (p=0.013 and p=0.017). Endocrine sexual symptoms, driven mainly by vaginal dryness and discomfort, were lowest at ≤39 years and highest at 50-59 years (p<0.001 versus both ≤39 and 40-49 years). Body image, endocrine therapy symptoms, and endocrine sexual symptoms also remained significant after Holm correction. Welch's ANOVA, which does not assume equal variances, confirmed the age differences for fatigue, body image, future perspective, breast symptoms, endocrine therapy symptoms, and endocrine sexual symptoms (all p≤0.034); it was borderline for arm symptoms (p=0.054) and could not be computed for appetite loss because one stratum had zero variance. Systemic therapy side effects differed on Kruskal-Wallis testing (q=0.034) but not on ANOVA after correction (q=0.071), and breast satisfaction and skin mucositis symptoms did not differ by age.

Sexual activity differed sharply by age (exact Monte Carlo p<0.001). All 11 women aged ≤39 years (100%) and 10 of 19 aged 40-49 years (52.6%) reported being sexually active, compared with none of the 21 women aged 50-59 years, 15 aged 60-69 years, or 4 aged ≥70 years; overall, 21 of 70 women (30.0%) were sexually active. Scored according to the EORTC manual, sexual functioning was therefore 0 in every woman aged 50 years or above, and it was higher at ≤39 than at 40-49 years (43.94±15.41 vs 19.30±17.80; Mann-Whitney p=0.002). Sexual enjoyment, scored only for the 21 sexually active women, was similar at ≤39 and 40-49 years (39.39±13.48 vs 36.67±10.54; p=0.64). These two scales were reported descriptively and not tested across all five strata. Breast satisfaction is reported for completeness, though its interpretability is limited in a cohort with no breast conservation or reconstruction.

Associations with clinical variables

In multivariable linear regression (Table 4), age remained independently associated with all eight scales that differed by age after adjustment for stage, neoadjuvant chemotherapy, and radiotherapy. Each 10-year increase in age was associated with better body image (β=6.88, 95% CI 4.93 to 8.83, p<0.001) and future perspective (β=5.67, 1.96 to 9.37, p=0.003), and with higher endocrine sexual symptoms (β=8.02, 4.75 to 11.30, p<0.001), endocrine therapy symptoms (β=4.06, 1.88 to 6.25, p<0.001), arm symptoms (β=3.47, 0.65 to 6.30, p=0.017), appetite loss (β=3.31, 0.40 to 6.22, p=0.027), breast symptoms (β=3.14, 0.27 to 6.02, p=0.033), and fatigue (β=3.61, 0.02 to 7.20, p=0.049). Stage III-IV disease was independently associated with higher fatigue, appetite loss, arm, breast, and endocrine therapy symptoms, and with lower global health status (β=-8.29, p=0.004) and QLQ-C30 summary score (β=-10.07, p=0.001), whereas age was not independently associated with either global measure. Neoadjuvant chemotherapy was independently associated with higher appetite loss and arm symptoms and a lower summary score. Pain was associated with stage (β=21.65, p<0.001) but not with age after adjustment (p=0.396). In unadjusted analyses, stage III-IV disease and neoadjuvant chemotherapy were associated with poorer scores on most key scales after FDR correction; radiotherapy was associated with higher arm symptoms, breast symptoms, and pain; longer hospital stay was associated with poorer body image (Spearman ρ=-0.39, q=0.004) and higher arm symptoms (ρ=0.30, q=0.033); and receptor group was associated with arm symptoms (q=0.033). Southampton wound grade was not associated with any scale after correction. Because 98.6% of patients reported severe pain on the day of surgery, VAS score could not be meaningfully analysed as a predictor.

**Table 4:** Multivariable linear regression of QoL scales on age, stage, and treatment (n=70) β: regression coefficient in points on the 0-100 scale. Each model includes age (per 10 years), stage (III-IV vs. I-II), neoadjuvant chemotherapy (NACT), and radiotherapy; all patients received adjuvant chemotherapy. Positive β indicates a higher score (better functioning for body image, future perspective, global health status, and summary score; greater burden for symptom scales). R^2^ indicates the proportion of variance explained.

Scale	Age per 10 years, unadjusted β (p)	Age per 10 years, adjusted β (95% CI)	p	Stage III-IV, β (p)	NACT, β (p)	Radiotherapy, β (p)	R^2^
Fatigue	5.30 (0.008)	3.61 (0.02 to 7.20)	0.049	14.17 (0.008)	7.99 (0.084)	3.88 (0.360)	0.33
Appetite loss	4.38 (0.008)	3.31 (0.40 to 6.22)	0.027	9.27 (0.031)	11.57 (0.003)	1.53 (0.657)	0.36
Body image	6.75 (<0.001)	6.88 (4.93 to 8.83)	<0.001	-0.91 (0.746)	-4.82 (0.055)	-1.16 (0.613)	0.48
Future perspective	5.01 (0.007)	5.67 (1.96 to 9.37)	0.003	-5.19 (0.334)	-4.96 (0.295)	-2.91 (0.505)	0.17
Arm symptoms	5.03 (0.004)	3.47 (0.65 to 6.30)	0.017	12.91 (0.002)	11.01 (0.003)	4.55 (0.175)	0.46
Breast symptoms	4.93 (0.003)	3.14 (0.27 to 6.02)	0.032	14.61 (<0.001)	4.76 (0.196)	5.44 (0.112)	0.40
Endocrine therapy symptoms	5.05 (<0.001)	4.06 (1.88 to 6.25)	<0.001	7.89 (0.014)	0.87 (0.753)	3.31 (0.200)	0.36
Endocrine sexual symptoms	8.66 (<0.001)	8.02 (4.75 to 11.30)	<0.001	4.89 (0.303)	-0.10 (0.980)	2.99 (0.439)	0.34
Pain	3.86 (0.043)	1.29 (-1.73 to 4.30)	0.396	21.65 (<0.001)	6.01 (0.121)	5.17 (0.148)	0.47
Global health status/QoL	-1.07 (0.300)	-0.17 (-2.11 to 1.76)	0.858	-8.29 (0.004)	-4.45 (0.074)	0.96 (0.674)	0.24
QLQ-C30 summary score	-2.61 (0.031)	-1.48 (-3.50 to 0.53)	0.147	-10.07 (<0.001)	-8.05 (0.002)	-0.45 (0.848)	0.42

USAQ scores

The USAQ was analysed separately from the EORTC instruments because it captures a comparatively stable, trait-like construct (unconditional self-acceptance in the context of illness) rather than the treatment or symptom-related burden that drives most of the QLQ-C30 and QLQ-BR45 variation described above. Age-wise USAQ scores are set out in Figure 1.

**Figure 1:**
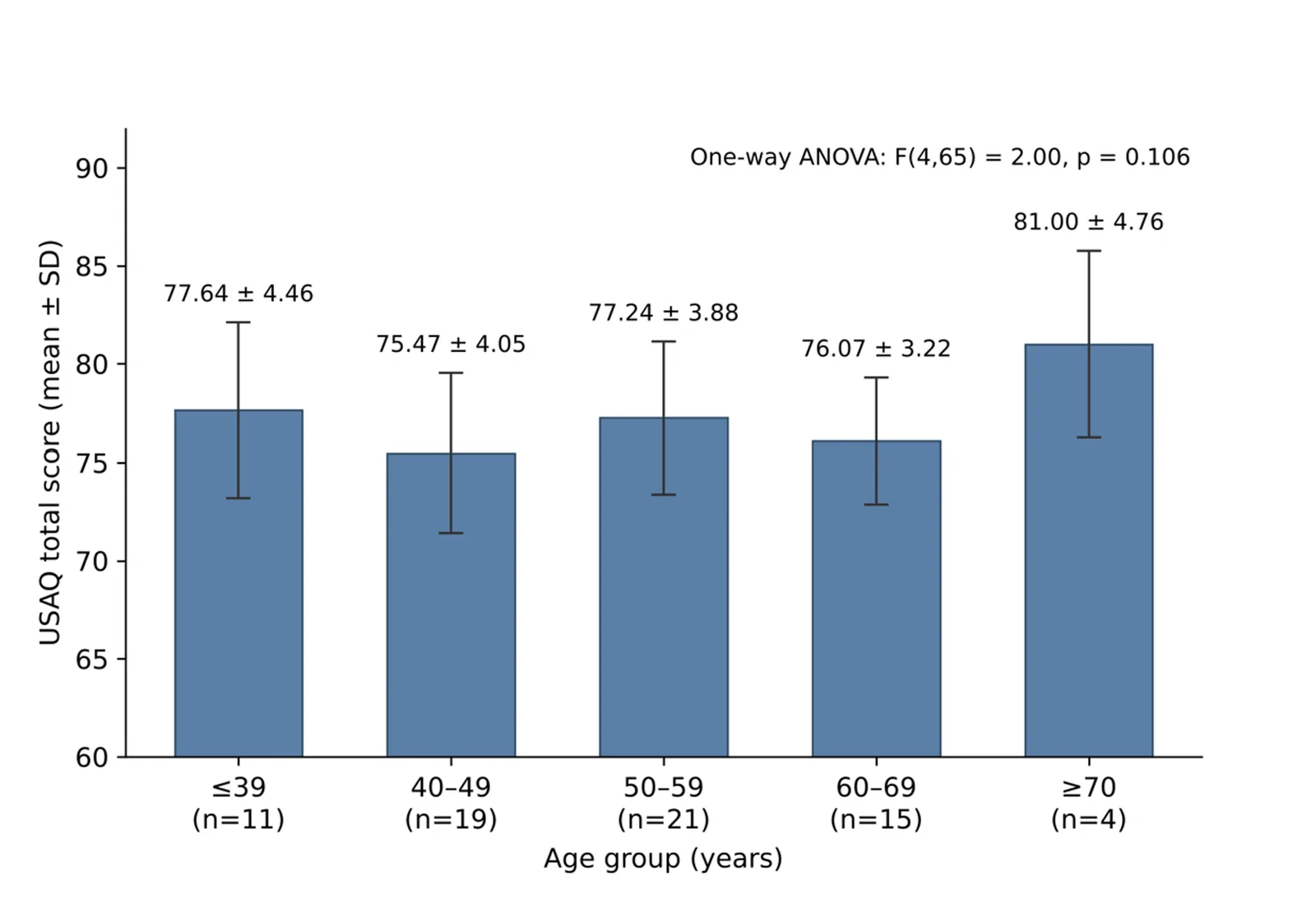
USAQ total scores across age groups, N=70 Bars show the mean ± SD of the USAQ total score (range 20-140). Values above each bar are mean ± SD; the y-axis starts at 60. USAQ scores are reverse-scored per the original scoring instructions of Chamberlain and Haaga [8], such that higher scores indicate greater unconditional self-acceptance. The total score did not vary significantly across age strata (one-way ANOVA, F(4,65)=2.00, p=0.106). USAQ: Unconditional Self-Acceptance Questionnaire; SD: standard deviation; ANOVA: analysis of variance

The USAQ total score did not vary significantly across the five age strata (one-way ANOVA, F(4,65)=2.00, p=0.106, η^2^=0.11, q=0.141; Kruskal-Wallis p=0.225), in contrast to the multiple significant age gradients seen on the QLQ-BR45. This relative age invariance is consistent with what the USAQ is designed to measure: a self-acceptance disposition that need not track the physical or body image burden of surgery.

## Discussion

This prospective study characterised QoL after MRM in 70 women using the EORTC QLQ-C30 and QLQ-BR45, complemented by the USAQ. Three findings stand out. First, overall QoL was moderate despite substantial early surgical morbidity. Second, the generic QLQ-C30 showed robust age variation only in fatigue and appetite loss, whereas its functional scales and global score did not differ significantly after correction. Third, the breast-specific QLQ-BR45 revealed a richer, age-associated pattern involving body image, future perspective, sexual activity, and several treatment-related symptom domains that would have been largely invisible on the QLQ-C30 alone, and these associations persisted after adjustment for stage and treatment.

The moderate summary scores observed here echo other survivorship cohorts in which mastectomy patients report average-to-good overall functioning yet persistent deficits in specific domains. Rose et al., in a hospital-based Indian study, similarly found preserved overall functioning but lower sexual functioning, body image, and future perspective scores among mastectomy survivors [20]. Comparable patterns of domain-specific impairment after mastectomy have been reported by Cherian et al. across serial follow-up [21], by Türk and Yılmaz in stage II-III survivors with marked body image impairment [22], and by Bhat et al., who described greater household activity limitation and feelings of incompleteness after MRM than after breast conservation [23]. Recent Indian series using the QLQ-BR45, including that of Nair et al., likewise emphasise that low functional-domain scores rather than symptom scores alone identify the most pressing targets for intervention [24].

Women aged 49 years or younger reported significantly poorer body image than older women, and this association persisted after adjustment for stage and treatment. This accords with Elmas et al., who found poorer vitality and mental-health outcomes among women under 50 undergoing MRM [25], and with the observation of Diao et al. that age materially influences long-term QoL [26]. Younger patients may experience breast loss as a greater threat to body image, sexual identity, and occupational and social roles, while treatment-induced endocrine and menopausal symptoms further compromise sexual health [27]. Sexual activity showed a striking age gradient: all women aged ≤39 years and about half of those aged 40-49 years were sexually active, but none of the 40 women aged 50 years or above were sexually active. Lower reported sexual activity among older Indian women, together with cultural reluctance to discuss it, may contribute to this pattern and limit the usefulness of the sexual scales in this setting. The absence of reported sexual activity should not be read as an absence of sexual health needs: endocrine sexual symptoms such as vaginal dryness were highest in women aged 50-69 years, and sexual health and body image should still be assessed and addressed in older women after MRM. Longitudinal comparisons of reconstruction with mastectomy alone suggest that these age-related vulnerabilities can be partly mitigated in appropriately selected younger, active women [28,29], although reconstruction was not part of the present MRM-only cohort. Conversely, the heavier symptom burden in women aged 50 years and above (fatigue, appetite loss, and arm, breast, and endocrine-therapy symptoms) persisted after adjustment for stage and treatment, which suggests a contribution from age-related factors such as menopausal status and comorbidity; these were not recorded separately and could not be examined directly.

Early postoperative morbidity was a prominent feature: severe pain affected 98.6% (n=69) of patients on the day of surgery, and the mean hospital stay exceeded 13 days. Post-mastectomy pain is a well-recognised driver of impaired QoL; Beyaz et al. documented its high prevalence and adverse QoL impact [4]. The prolonged length of stay in the present cohort reflects institutional practice of retaining patients until drain output fell below the removal threshold and the wound was reviewed, rather than a uniformly complicated postoperative course. These observations are consistent with evidence that structured analgesia and early, protocolised rehabilitation improve functional and symptom outcomes after mastectomy [30], although the present study did not evaluate these interventions. The consistently high financial difficulty scores on the QLQ-C30, despite treatment in a public facility, highlight a non-clinical dimension of survivorship that also merits attention. Disease stage was the strongest clinical correlate of QoL in this cohort: stage III-IV disease was independently associated with poorer global health status and summary score and with a higher symptom burden, which underlines that oncological factors and QoL are closely linked at every age.

More age differences were seen on the QLQ-BR45 than on the generic QLQ-C30, but this reflects its content rather than demonstrated greater sensitivity: the QLQ-BR45 contains breast-specific domains, such as body image and arm and endocrine therapy symptoms, that the QLQ-C30 does not measure. These domains are frequently among those most affected after mastectomy, which supports using the two instruments together [7]. The relative age invariance of USAQ scores adds a complementary perspective: unconditional self-acceptance behaved as a stable trait not strongly modulated by age at six months after surgery, suggesting that psychological adjustment and symptom or body image burden are partly independent constructs and that both warrant assessment [8]. Taken together, the findings argue for QoL evaluation that is domain-specific and age-aware rather than reliant on a single global index.

Limitations

This study has several limitations. It was conducted at a single centre with a modest sample of 70 patients, which constrained subgroup analysis, most notably in the ≥70-year stratum (n=4). No a priori sample-size calculation was performed, and the sensitivity analysis indicates that only large age effects could be reliably detected; non-significant findings, including global health status, may therefore be false negatives. All participants underwent MRM, so no internal comparison with breast-conserving surgery or reconstruction was possible. Randomisation between surgical procedures is not feasible because the choice of MRM is driven by tumour factors and patient preference; a prospective comparative study including breast-conserving surgery and reconstruction would be the appropriate next step. Age comparisons were cross-sectional and cannot separate age effects from cohort effects. The QoL questionnaires were completed at the six-month follow-up visit, but the exact interval from surgery for each patient and adjuvant treatment status at that time were not analysed, and women still receiving treatment may have reported higher symptom scores. Within-patient change from diagnosis was not analysed, so the change attributable to surgery itself cannot be quantified. Although the main age associations were adjusted for stage, neoadjuvant chemotherapy, and radiotherapy, menopausal status and comorbidity were not recorded separately, so residual confounding is possible. Endocrine therapy status at the six-month visit was not analysed, and endocrine symptoms may partly reflect this treatment rather than age. The English questionnaires (EORTC and USAQ) were translated verbally by the authors for women who could not read English, and these translations were not formally validated. Several EORTC domains showed pronounced floor or ceiling effects, and sexual functioning could not be compared statistically across all age groups because no woman aged 50 years or above was sexually active. The QLQ-C30 findings for nausea and vomiting, pain, and constipation did not survive correction for multiple comparisons. The age-related findings should therefore be regarded as hypothesis-generating. Larger, multicentre, longitudinal studies with comparator surgical arms and within-patient analysis of change from baseline would help confirm the patterns observed here.

## Conclusions

Oncological clearance remains the primary goal of MRM at every age. Alongside it, QoL after MRM in this single-centre cohort was multidimensional and associated with age and disease stage. The breast-specific EORTC QLQ-BR45 identified age-associated differences in body image, future perspective, sexual activity, and treatment-related symptoms that the generic QLQ-C30 largely did not, with younger women reporting poorer body image and older women a heavier symptom burden, and these differences persisted after adjustment for stage and treatment. Advanced stage was the strongest clinical correlate of poorer global QoL. Together with the high rate of severe early pain and prolonged hospitalisation, these findings suggest that pain control, arm morbidity, body image, sexual health, and psychological needs may warrant attention in survivorship care. Because the findings are exploratory, they need confirmation in larger, adequately powered studies, and interventions targeting these domains should be evaluated in future research.
